# Mental health service use by recent immigrants from different world regions and by non-immigrants in Ontario, Canada: a cross-sectional study

**DOI:** 10.1186/s12913-015-0995-9

**Published:** 2015-08-20

**Authors:** Anna Durbin, Rahim Moineddin, Elizabeth Lin, Leah S. Steele, Richard H. Glazier

**Affiliations:** 1 Canadian Mental Health Association (Toronto branch), Toronto, Canada; 2 Department of Family and Community Medicine, University of Toronto, Toronto, Canada; 3 Institute for Clinical Evaluative Sciences, Toronto, Canada; 4 Department of Psychiatry, University of Toronto, Toronto, Canada; 5 Provincial System Support Program, Centre for Addiction and Mental Health, Toronto, Canada; 6 Keenan Research Centre in the Li Ka Shing Knowledge Institute at St. Michael’s Hospital, Toronto, Canada; 7 Institute of Health Policy, Management and Evaluation, University of Toronto, Toronto, Ontario Canada

## Abstract

**Background:**

Given that immigration has been linked to a variety of mental health stressors, understanding use of mental health services by immigrant groups is particularly important. However, very little research on immigrants’ use of mental health service in the host country considers source country. Newcomers from different source countries may have distinct experiences that influence service need and use after arrival. This population study examined rates of use of primary care and of specialty services for non-psychotic mental health disorders by immigrants to Ontario Canada during their first five years after arrival. Service use by recent immigrants in broad source region groups representing all world regions was compared to use by age-matched Canadian-born or long term immigrants (called long term residents).

**Method:**

This matched population-based cross-sectional study assessed likelihood of any use and counts of visits for each of primary care, psychiatric care and hospital care (emergency department visits or inpatient admissions) for non-psychotic mental health disorders from 1993–2012. Adult immigrants living in urban Ontario (*n* = 912,114) were categorized based on their nine world regions of origin. Sex-stratified conditional logistic regression models and negative binomial models were used to compare service use by immigrant region groups to their age-matched long term residents.

**Results:**

Immigrant were more or less likely to access primary mental health care compared to age-matched long term residents, depending on their world region of origin. Regarding specialty mental health care (psychiatry and hospital care), immigrants from all regions used less than long term residents. Across the three mental health services, estimates of use by immigrant region groups compared to long term residents were among the lowest for newcomers from East Asian and Pacific (range: 0.16–0.82) and among the highest for persons from Middle East and North Africa (range: 0.56–1.23).

**Conclusion:**

This population-based study showed lower use of mental health services by recent immigrants than long-term immigrants or native born individuals, with variation in immigrants’ use linked to world region of origin and type of mental health care. Variation across source region groups underscores the importance of identifying underlying individual characteristics that affect service use to make services more responsive to newcomers.

**Electronic supplementary material:**

The online version of this article (doi:10.1186/s12913-015-0995-9) contains supplementary material, which is available to authorized users.

## Background

As the number of immigrants grows worldwide, [1] so does the attention to immigrants’ use of health and social services [2, 3]. However, there is limited research on current service use patterns to inform efforts to improve quality of care. Moreover, existing research rarely accounts for the diversity of immigrant populations, [4] such as that driven by world region of origin. Immigration is increasingly a global phenomenon and newcomers from varied regions often have distinct pre-immigration experiences (i.e., social, cultural, and political), as well as varied post migration re-settlement experiences [5–7] that may influence both service need, and factors that assist or impede access to care [7–9].

Given that immigration has been linked to a variety of mental health stressors, [7, 8, 10] understanding use of mental health services by immigrant groups is particularly important [11, 12]. While both the pre and post immigration context can influence health and help seeking, [8, 10, 13] research rarely accounts for immigrant source region. In fact, only three mental health service use studies examined immigrants from different source countries. Of these, two studies were Dutch that showed variation by source country. One by Selten and colleagues [14] showed lower use for care for mood disorders by immigrants from Turkey, Morocco, and Surinam than by native born Dutch as well as variation across the three source country immigrant groups [14]. Suggested explanations for the variation included group differences in thresholds for seeking treatment, familiarity with pathways to psychiatric care, and likelihood of referrals by clinicians. The other Netherlands based study by Uiters and others [15] examined primary and specialty mental health care utilization by immigrants from Turkey, Surinam, Morocco, and The Netherlands Antilles. Compared to indigenous people, newcomers from Morocco were less likely to use a combination of primary care and mental health services while people from the Netherlands Antillean were more likely to use these forms of care [15]. The authors suggested that differences in use among immigrant groups may reflect their experience with service delivery in their home countries, particularly the role of primary care in facilitating access to speciality mental health services. A Canadian study [16] on immigrants from the Caribbean, Vietnam, the Philippines living in Montreal, Canada found that Vietnamese and Filipino immigrants were one-third as likely as Canadian-born residents to use mental health care, although there were no differences between Caribbean newcomers and Canadian-born.

Regarding use of hospital services for non-psychotic disorders, studies have shown more use by immigrants compared to native born persons; [17–19] less use; [20] or no differences in use, [21, 22] but have not disaggregated by world region of origin.

In the context of global immigration, profiling mental health service use by newcomers’ source countries can provide useful information. Existing studies of this type are sparse and have looked at specific source country groups. Investigating patterns across an entire immigrant population in the same setting and with respect to a common comparator can provide a more comprehensive picture. The underlying reasons for any distinct use patterns that are observed may then be further investigated.

We sought to contribute to existing knowledge by examining mental health service use by immigrants from the full range of regions in a large, diverse province with a single payer health care system. Ontario, Canada is a major destination for immigrants where 27 % of the population is foreign-born with source countries from almost every continent [23]. This study compared rates of primary care visits, psychiatry visits and hospital use for non-psychotic mental health disorders for recent immigrants to Ontario from nine world regions of origin to long term residents (LTRs), a group of long term immigrants or Canadian born individuals to whom immigrants were matched on age.

## Methods

This population-based cross-sectional study was conducted using linked administrative data in Ontario, Canada. Access to study data was possible through a comprehensive research agreement with Ontario’s Ministry of Health and Long-Term Care. The research protocol was approved by Research Ethics Boards at the University of Toronto and Sunnybrook Health Sciences Centre in Toronto.

### Data sources

Several databases were linked using unique, encoded identifiers and analyzed at the Institute for Clinical Evaluative Sciences (ICES). The Ontario portion of the Citizenship and Immigration Canada (CIC) database contains individual-level demographic information recorded at landing for Ontario’s permanent residents who arrived from 1985 to 2010. In addition to demographic data, it includes country of birth, source country, admission category, education level, marital status, official language speaking ability and year of arrival. The Registered Persons Database (RPDB) is Ontario’s health care registry, and includes age, sex, and postal codes of all Ontario residents who are eligible for the province’s single universal health care plan, the Ontario Health Insurance Plan (OHIP). An initial validation study [24] of the linkage between the Ontario CIC and RPDB found that 84.4 % of records in the CIC were successfully linked. OHIP insures medically necessary care delivered by physicians and in hospital settings without user fees, co-payments or deductibles [25]. Eligibility for OHIP for immigrants begins after they have resided in Ontario for three months, but for refugees this wait period is more variable [26]. OHIP claims data from 1993 to 2012 on primary mental health care and psychiatry care were categorized by type of provider visited based on OHIP specialty code. Mental health admissions were determined from the Canadian Institute for Health Information’s Discharge Abstract Database (1993–2012) and the Ontario Mental Health Reporting System (2005/6-2012). Mental health emergency department (ED) visits were determined from variables from the OHIP claims data that identified services delivered in the ED (1993–2001), and the National Ambulatory Care Reporting System (2002–2012). We used Statistics Canada’s Postal Code Conversion File to link patients’ postal codes to census data to determine urban residence and neighbourhood income quintiles associated with their dissemination area [27, 28]. (For more details, see Additional file 1).

### Study populations

The initial sample included 1,618,672 immigrants listed in the Ontario CIC who arrived to Ontario from April 1, 1993 and March 31, 2007. This period was selected since full health service use records in Ontario were available from April 1, 1993 until March 31, 2012, allowing for five year follow-up from all eligible arrival dates. We then applied further sample inclusion criteria: being aged 18–105 years for the 5-year outcome window within the study period (1993–2012), having OHIP coverage, having at least one contact with the health care system during the outcome window, and living in metropolitan areas in Ontario. Rural populations were excluded because 98 % of immigrants in this database settled in urban areas [29]. Imposing these inclusion criteria left a sample of 971,758 eligible immigrants. Final exclusions were based on immigrant characteristics. We excluded those who did not immigrate to Canada directly from their birth country (i.e., their country of birth was different the country from which they immigrated), whose country of origin could not be classified, or who were admitted in the ‘other’ admission class (i.e., were not admitted in the economic, family, or refugee classes). After these exclusions, the study sample included 912,114 immigrants. In total, 99.6 % of these immigrants were matched to LTRs on sex and birthdate at a ratio of 1:1.

LTRs were Canadian-born individuals or newcomers who settled in Ontario prior to 1985. We applied similar inclusion criteria to LTRs as those used for immigrants: 18 years or older, residence in urban areas, and OHIP eligibility during the study period. To avoid misclassifying immigrants who are not included in the Ontario CIC as LTRs, we also excluded adults who were not in the CIC and first became eligible for OHIP after 1993. Newcomers may be absent from the Ontario CIC if they initially declared an intention to move to another Canadian province but ultimately moved to Ontario, or if they could not be probabilistically linked to RPDB [29, 30].

### Independent variables

#### Sex

Analyses were stratified by sex because females are more likely than males to experience non-psychotic mental health disorders (e.g., depression) and use mental health services [31–33]. There is also evidence that immigration related factors, such as world region of origin, are associated with mental health disorders in different ways for males and females [34, 35].

#### Age

Immigrants were matched to LTRs on exact birthdate because age is related to mental health need and service use [31, 32].

#### Region of origin

Immigrants were categorized into nine mutually exclusive regions of origin based on their source countries listed in the CIC. Groupings were based on a modified version of the United Nations Children’s Fund (UNICEF) classification system that has been used in a growing body of immigrant research: industrialized countries, Central and Eastern Europe, Middle East and North Africa, Eastern and Southern Africa, Latin America, the Caribbean, South Asia, West and Central Africa, and East Asia and the Pacific (See Additional file 2 for classification of specific countries) [36]. One adaptation from the UNICEF classification was that the Latin America and the Caribbean category was separated into two categories: Latin America and Caribbean.

#### Income quintile

Neighbourhood income quintile was included as a covariate in the adjusted analysis because immigrants are over-represented in disadvantaged areas [8, 37]. In turn, most studies have shown that living in these disadvantaged areas has been linked to lower access to outpatient specialty mental health care, even in publically funded systems where patients experience fewer financial barriers to use of mental health services [38–40].

#### Immigration variables (descriptive analysis)

Immigration variables were determined from the CIC. Individual-level demographic information is recorded at landing for Ontario’s permanent residents who landed from 1985 to 2010. In addition to demographic data, the study included source country, country of birth, admission category, education level, marital status, official language speaking ability, and year of arrival.

### Service use outcomes

Three mental health service use outcomes were measured for immigrants and their matched LTRs during the same five years that followed the start of the immigrant’s eligibility for OHIP: 1) visits to primary care physicians, 2) visits to psychiatrists, and 3) a composite of ED visits or hospital admissions. Short-term admissions (i.e., admissions of 72 h or less) were excluded because the information used to classify conditions for which services were sought did not allow for the distinction between non-psychotic and psychotic disorders. Our method for identifying nonpsychotic primary care visits (using codes in Additional file 3) has been used in previous studies and shown a sensitivity of 81 % and a specificity of 97 % for identifying mental health visits to primary care physicians [41, 42]. To include hospital visits in which the underlying problem is a mental health issue, we broadly defined mental health ED visits and hospital admissions as admissions for which any diagnosis field was related to non-psychotic mental disorders based on International Classification of Disease codes (See Additional file 3).

### Statistical analysis

#### Descriptive analyses

Demographic and immigration characteristics were calculated for immigrants across the nine world regions, stratified by sex. T-tests and chi-square tests were used to examine the statistical significance of differences across region groups. In addition, in an unadjusted analysis we examined use of each type of mental health care (primary mental health care, psychiatric care, hospital mental health care) for immigrants by region, and for LTRs. Analyses were conducted using SAS version 9.3 (SAS Institute Inc., Cary, NC, USA).

#### Adjusted analyses

For each outcome we modelled access (i.e., any use of services) and intensity of service use (i.e., counts of use) during the five-year outcome window. We modelled access using conditional logistic regression [43] and utilization among those with any access using negative binomial models with Generalized Estimating Equations. These models were used because they accounted for the outcome types (binary and counts respectively), and were suited to the matched nature of the data [44].

Models of mental health care use were stratified by immigrant world region of origin and sex, and adjusted for neighbourhood income quintile. Characteristics that applied to immigrants and not LTRs (e.g., admission class) could not be included in the adjusted models since the information collected from immigrants at landing was not available or relevant for LTRs. Estimates of use for each immigrant region group compared to their matched LTRs were presented on forest plots. Results from models of intensity of utilization among the entire sample are not shown as they yielded results similar to intensity of utilization models among persons with any care use.

In the primary analysis hospital use was categorized as a mental health admission if any diagnosis field included a non-psychotic mental health disorder. Since this potentially included hospitalizations not driven by mental health problems, a sensitivity analysis was conducted where mental health hospital use only included uses for which the most responsible diagnosis was for a non-psychotic mental health disorder.

## Results

### Descriptive characteristics for immigrants by world region of origin and sex

Of all newcomers (males: *n* = 422,373, 46.3 %; females: *n* = 489,741, 53.7 %), nearly half were from South Asia (30.1 % of males; 27.4 % of females), or East Asia and the Pacific (21.0 % of males; 25.1 % of females) (Tables 1 and 2). As indicated in the Table 1, all characteristics varied among immigrants from the nine world region of origins (*p* < 0.001). Those from Western and Central Africa were youngest (males: 33.60 years; females: 32.8 years). Regarding admission class, immigrants from Central and Eastern Europe were most often admitted in the economic admission class (males: 52.9 %; females: 56.1 %), immigrants from Caribbean were mostly admitted in the family reunification class (males: 45.2 %; females: 62.3 %), and those from East and Southern Africa were most commonly admitted as refugees (males: 57.4 %; females: 53.3 %). Immigrants from Central and Eastern Europe and from South Asia were most likely to be married (Central and Eastern European males: 72.8 %; South Asian females: 78.5 %), while the Central and Eastern Europe group was also most likely to have a more than a high school education (males: 77.8 %; females: 72.7 %). The proportion who spoke English or French was highest for immigrants from the Caribbean (males: 99.5 %; females: 99.3 %). Immigrants from West and Central Africa were most commonly in the lowest income quintile (males: 51.9 %; females: 54.2 %); LTRs were under-represented in this quintile (both sexes: 18.3 %). Immigrants from industrialized countries were most commonly in the most affluent income quintile (males: 19.8 % females: 19.3 %, Tables 1 and 2).

**Table 1:** Characteristics for recent adult male immigrants who arrived in Ontario from 1993–2007 (18 years+), by region of origin, and for their matched long term resident comparators in urban Ontario (N (%))

		Immigrants by region^c^											Matched comparators
Characteristics	Central and Eastern Europe	Caribbean	East Asia and Pacific	Eastern and Southern Africa	Latin America	Industrialized countries	Middle East and North Africa	South Asia	West and Central Africa	All immigrants	*P*-value*	Long term residents^d^	
Population	N (%)	41,996 (9.9)	27,459 (6.5)	91,248 (21.0)	12,094 (2.9)	20,306 (4.81)	56,601 (13.4)	36,643 (8.7)	127,110 (30.1)	8,916 (2.1)	422,373 (100.0)		420,578 (99.6 %)
Age at immigration (years)^a^	Mean ± SD	36.24 ± 11.11	34.93 ± 12.01	37.42 ± 12.52	33.73 ± 11.97	34.49 ± 10.51	36.53 ± 11.59	36.34 ± 12.17	36.22 ± 12.58	33.60 ± 9.39	36.24 ± 12.09	<.001	
Admission Class^a^	Economic	28,284 (67.3)	8,987 (32.7)	55,251 (60.6)	3,044 (25.2)	8,015 (39.5)	32,530 (57.5)	19,325 (52.7)	64,346 (50.6)	3,668 (41.1)	223,450 (52.9)	<.001	
	Family	5,440 (13.0)	18,135 (66.0)	30,413 (33.3)	2,100 (17.4)	6,543 (32.2)	21,226 (37.5)	6,140 (16.8)	42,273 (33.3)	2,624 (29.4)	134,894 (31.9)		
	Refugees	8,272 (19.7)	337 (1.2)	5,584 (6.1)	6,950 (57.4)	5,748 (28.3)	2,845 (5.0)	11,178 (30.5)	20,491 (16.1)	5,748 (28.3)	2,624 (29.4)		
Marital Status^a^	Married	30,563 (72.8)	17,628 (64.2)	65,982 (72.3)	6,256 (51.7)	13,488 (66.4)	39,533 (69.8)	20,416 (55.7)	78,365 (61.7)	4,915 (55.1)	277,146 (65.6)	<.001	
	Separated	1,438 (3.4)	824 (3.0)	1,467 (1.6)	346 (2.9)	679 (3.3)	1,566 (2.8)	706 (1.9)	1,742 (1.4)	281 (3.2)	9,049 (2.1)		
	Single	9,983 (23.8)	8,971 (32.7)	23,784 (26.1)	5,491 (45.4)	6,127 (30.2)	15,494 (27.4)	15,509 (42.3)	46,980 (37.0)	3,714 (41.7)	136,053 (32.2)		
Education level^a^	None	226 (0.5)	278 (1.0)	975 (1.1)	394 (3.3)	305 (1.5)	472 (0.8)	437 (1.2)	3,374 (2.7)	82 (0.9)	6,543 (1.5)	<.001	
	More than high school	32,659 (77.8)	7,737 (28.2)	64,999 (71.2)	5,444 (45.0)	12,402 (61.1)	36,139 (63.8)	23,692 (64.7)	80,081 (63.0)	5,490 (61.6)	268,643 (63.6)		
	Secondary	9,111 (21.7)	19,444 (70.8)	25,274 (27.7)	6,256 (51.7)	7,599 (37.4)	19,990 (35.3)	12,514 (34.2)	43,655 (34.3)	3,344 (37.5)	147,187 (34.8)		
Language^a^	English/French	29,471 (70.2)	27,313 (99.5)	46,754 (51.2)	10,707 (88.5)	15,180 (74.8)	44,797 (79.1)	28,254 (77.1)	94,501 (74.3)	8,405 (94.3)	305,382 (72.3)	<.001	
	Neither	12,524 (29.8)	144 (0.5)	44,494 (48.8)	1,387 (11.5)	5,126 (25.2)	11,804 (20.9)	8,389 (22.9)	32,607 (25.7)	510 (5.7)	116,985 (27.7)		
Period of arrival^a^	1993–1997	14,108 (33.6)	14,294 (52.1)	25,286 (27.7)	4,478 (37.0)	6,035 (29.7)	26,897 (47.5)	12,432 (33.9)	34,126 (26.8)	2,387 (26.8)	140,043 (33.2)	<.001	
	1998–2002	17,429 (41.5)	7,778 (28.3)	34,923 (38.3)	3,460 (28.6)	6,194 (30.5)	16,452 (29.1)	13,084 (35.7)	50,119 (39.4)	3,271 (36.7)	152,710 (36.2)		
	2003–2007	10,459 (24.9)	5,387 (19.6)	31,039 (34.0)	4,156 (34.4)	8,077 (39.8)	13,252 (23.4)	11,127 (30.4)	42,865 (33.7)	3,258 (36.5)	129,620 (30.7)		
Area income quintile^b^	1 (low)	18,988 (45.2)	12,287 (44.7)	33,470 (36.7)	5,803 (48.0)	7,533 (37.1)	11,886 (21.0)	14,247 (38.9)	56,150 (44.2)	4,630 (51.9)	164,994 (39.1)	<.001	77,043 (18.3)
	2	9,013 (21.5)	6,690 (24.4)	25,082 (27.5)	2,227 (18.4)	4,899 (24.1)	11,564 (20.4)	7,368 (20.1)	30,768 (24.2)	1,979 (22.2)	99,590 (23.6)		84,172 (20.0)
	3	5,703 (13.6)	4,357 (15.9)	15,615 (17.1)	1,474 (12.2)	3,191 (15.7)	10,903 (19.3)	5,988 (16.3)	20,917 (16.5)	1,121 (12.6)	69,269 (16.4)		85,744 (20.4)
	4	4,347 (10.4)	2,653 (9.7)	10,009 (11.0)	1,215 (10.0)	2,445 (12.0)	10,294 (18.2)	4,843 (13.2)	12,267 (9.7)	703 (7.9)	48,776 (11.5)		86,269 (20.5)
	5 (high)	3,531 (8.4)	1,176 (4.3)	6,163 (6.8)	1,128 (9.3)	1,948 (9.6)	11,231 (19.8)	3,564 (9.7)	5,712 (4.5)	344 (3.9)	34,797 (8.2)		83,801 (19.9)
	Missing	414 (1.0)	296 (1.1)	909 (1.0)	247 (2.0)	290 (1.4)	723 (1.3)	633 (1.7)	1,296 (1.0)	139 (1.6)	4,947 (1.2)		3,549 (0.8)

*T-tests and chi-square tests compared characteristics among immigrants from the CIC across nine mutually exclusive regions

^a^From the CIC

^b^Not from the CIC

^c^Immigrants who arrived in Canada between 1993 and 2007 with Ontario as their intended destination were identified in the Citizenship and Immigration Canada (CIC) database. Region groupings were based on a modified version of the United Nations Children’s Fund (UNICEF) classification system

^d^Long term residents were Canadian born or long term immigrants who arrived pre-1985

**Table 2:** Characteristics for recent adult female immigrants (18 years+) who arrived in Ontario from 1993–2007, by region of origin, and for their matched long term resident comparators in urban Ontario (N (%))

		Immigrants by region^c^											Matched comparators
Characteristics		Central and Eastern Europe	Caribbean	East Asia and Pacific	Eastern and Southern Africa	Latin America	Industrialized countries	Middle East and North Africa	South Asia	West and Central Africa	All immigrants	*P*-value*	Long term residents^d^
Population size	N (%)	47,967 (9.8)	32,726 (6.7)	122,711 (25.1)	15,529 (3.2)	23,673 (4.8)	69,137 (14.1)	35,445 (7.2)	134,029 (27.4)	8,524 (1.7)	489,741 (100.0)		487,751 (99.6 %)
Age at immigration (years)^a^	Mean ± SD	36.86 ± 12.84	36.36 ± 13.50	36.58 ± 12.60	34.04 ± 13.66	35.22 ± 11.92	36.59 ± 12.92	35.62 ± 12.99	35.04 ± 13.30	32.83 ± 10.32	35.89 ± 12.95	<.001	
Admission class^a^	Economic	26,908 (56.1)	11,610 (35.5)	67,760 (55.2)	2,993 (19.3)	7,209 (30.5)	32,742 (47.4)	14,743 (41.6)	45,913 (34.3)	2,858 (33.5)	212,736 (43.4)	<.001	
	Family	13,101 (27.3)	20,397 (62.3)	50,059 (40.8)	4,271 (27.5)	10,799 (45.6)	34,341 (49.7)	13,109 (37.0)	71,894 (53.6)	3,439 (40.3)	221,410 (45.2)		
	Refugees	7958 (16.6)	716 (2.2)	4892 (4)	8265 (53.3)	5665 (23.9)	2054 (3)	7593 (21.5)	16222 (12.1)	2227 (26.1)	64,029 (15.2)		
Marital Status^a^	Married	35,685 (74.4)	17,777 (54.3)	83,536 (68.1)	8,272 (53.3)	15,987 (67.5)	49,420 (71.5)	25,392 (71.6)	105,184 (78.5)	5,036 (59.1)	346,289 (70.7)	<.001	
	Separated	5,493 (11.5)	3,389 (10.4)	7,950 (6.5)	2,096 (13.5)	2,141 (9.0)	5,321 (7.7)	2,919 (8.2)	8,588 (6.4)	803 (9.4)	38,700 (7.9)		
	Single	6,777 (14.1)	11,528 (35.2)	31,210 (25.4)	5,158 (33.2)	5,537 (23.4)	14,388 (20.8)	7,127 (20.1)	20,243 (15.1)	2,681 (31.5)	104,649 (21.4)		
Education level^a^	None	450 (0.9)	493 (1.5)	2,040 (1.7)	1,411 (9.1)	556 (2.3)	1,044 (1.5)	1,260 (3.6)	9,616 (7.2)	277 (3.2)	17,147 (3.5)	<.001	
	More than high school	34,852 (72.7)	8,308 (25.4)	78,399 (63.9)	4,675 (30.1)	13,877 (58.6)	38,716 (56.0)	18,452 (52.1)	64,605 (48.2)	4,136 (48.5)	266,020 (54.3)		
	Secondary	12,665 (26.4)	23,925 (73.1)	42,272 (34.4)	9,443 (60.8)	9,240 (39.0)	29,377 (42.5)	15,733 (44.4)	59,808 (44.6)	4,111 (48.2)	206,574 (42.2)		
Language^a^	English/French	28,504 (59.4)	32,497 (99.3)	60,169 (49.0)	12,362 (79.6)	15,117 (63.9)	49,589 (71.7)	22,000 (62.1)	71,509 (53.4)	7,421 (87.1)	299,168 (61.1)	<.001	
	Neither	19,463 (40.6)	229 (0.7)	62,542 (51.0)	3,167 (20.4)	8,555 (36.1)	19,547 (28.3)	13,445 (37.9)	62,520 (46.6)	1,103 (12.9)	190,571 (38.9)		
Period of arrival^a^	1993–1997	15,078 (31.4)	17,199 (52.6)	37,141 (30.3)	5,659 (36.4)	6,548 (27.7)	34,400 (49.8)	10,601 (29.9)	35,498 (26.5)	2,101 (24.6)	164,225 (33.5)	<.001	
	1998–2002	19,679 (41.0)	9,396 (28.7)	42,307 (34.5)	4,848 (31.2)	7,364 (31.1)	19,400 (28.1)	12,544 (35.4)	49,073 (36.6)	2,952 (34.6)	167,563 (34.2)		
	2003–2007	13,210 (27.5)	6,131 (18.7)	43,263 (35.3)	5,022 (32.3)	9,761 (41.2)	15,337 (22.2)	12,300 (34.7)	49,458 (36.9)	3,471 (40.7)	157,953 (32.3)		
Income quintile^b^	1 (low)	20,878 (43.5)	14,251 (43.5)	43,097 (35.1)	7,935 (51.1)	8,688 (36.7)	14,901 (21.6)	13,601 (38.4)	58,547 (43.7)	4,619 (54.2)	186,517 (38.1)	<.001	89,042 (18.3)
	2	10,280 (21.4)	7,941 (24.3)	32,283 (26.3)	2,870 (18.5)	5,620 (23.7)	14,420 (20.9)	7,036 (19.9)	32,662 (24.4)	1,816 (21.3)	114,928 (23.5)		97,505 (20.0)
	3	6,579 (13.7)	5,324 (16.3)	20,668 (16.8)	1,759 (11.3)	3,699 (15.6)	13,236 (19.1)	5,902 (16.7)	22,277 (16.6)	1,020 (12.0)	80,464 (16.4)		99,135 (20.3)
	4	5,338 (11.1)	3,349 (10.2)	14,259 (11.6)	1,400 (9.0)	2,975 (12.6)	12,407 (17.9)	4,976 (14.0)	13,251 (9.9)	649 (7.6)	58,604 (12.0)		100,623 (20.6)
	5 (high)	4,468 (9.3)	1,562 (4.8)	11,156 (9.1)	1,326 (8.5)	2,421 (10.2)	13,357 (19.3)	3,498 (9.9)	6,160 (4.6)	314 (3.7)	44,262 (9.0)		97,952 (20.1)
	Missing	424 (0.9)	299 (0.9)	1,248 (1.0)	239 (1.5)	270 (1.1)	816 (1.2)	432 (1.2)	1,132 (0.8)	106 (1.2)	4,966 (1.0)		3,494 (0.7)

*T-tests and chi-square tests compared characteristics among immigrants from the CIC across nine mutually exclusive regions

^a^From the CIC

^b^Not from the CIC

^c^Immigrants who arrived in Canada between 1993 and 2007 with Ontario as their intended destination were identified in the Citizenship and Immigration Canada (CIC) database. Region groupings were based on a modified version of the United Nations Children’s Fund (UNICEF) classification system

^d^Long term residents were Canadian born or long term immigrants who arrived pre-1985

### Unadjusted analyses

Estimates of any use of primary mental health care varied among immigrant world region groups. Having any primary care use was most common for immigrants from West and Central Africa (males: 40.7 %; females: 52.9 %) and least common for immigrants from industrialized countries (males: 25.7 %) and East Asian and Pacific (females: 39.2 %) (Table 3). LTR’s estimates of any use primary mental health care (males: 30.9 %; females: 47.9 %) exceeded estimates for about one half of immigrant groups.

**Table 3:** Any mental health care use among adult recent immigrants and age-matched long term residents in Ontario, by immigrant world region of origin, sex, and type of mental health care, 1993–2012

	Immigrants by world region											Long term residents
		Central and Eastern Europe	Caribbean	East Asian and Pacific	Eastern and Southern Africa	Latin America	Industrialized countries	Middle East and North Africa	South Asia	West and Central Africa	Total	
Males												
	N	41,996	27,459	91,248	12,094	20,306	56,601	36,643	127,110	8,916	422,373	422,373
	(%)	9.9	6.5	21	2.9	4.8	13.4	8.7	30.1	2.1	100	100
Any mental health primary care use	N	11,674	9,516	24,939	4,044	7,041	14,523	13,324	41,119	3,627	129,807	129,932
	(%)	27.8	34.7	27.3	33.4	34.7	25.7	36.4	32.4	40.7	30.7	30.9
Any psychiatry use	N	1,290	676	955	452	893	1,791	2,118	3,131	217	11,523	23,965
	(%)	3.1	2.5	1.1	3.7	4.4	3.2	5.8	2.5	2.4	2.7	5.7
Any mental health hospital use	N	520	348	442	222	376	624	763	1,363	140	4,798	11,871
	(%)	1.2	1.3	0.5	1.8	1.9	1.1	2.1	1.1	1.6	1.1	2.8
Females												
	N	47,967	32,726	122,711	15,529	23,673	69,137	35,445	134,029	8,524	489,741	489,741
	(%)	9.8	6.7	25.1	3.2	4.8	14.1	7.2	27.4	1.7	100	100
Any mental health primary care use	N	21,499	16,473	48,056	7,189	12,320	27,379	17,688	57,402	4,510	212,516	233,641
	(%)	44.8	50.3	39.2	46.3	52	39.6	49.9	42.8	52.9	43.4	47.9
Any psychiatry use	N	2,274	1,028	1,833	619	1,562	3,036	2,685	3,500	227	16,764	36,863
	(%)	4.7	3.1	1.5	4.0	6.6	4.4	7.6	2.6	2.7	3.4	7.6
Any mental health hospital use	N	1,088	799	1,369	380	715	1,179	1,195	2,552	238	9,515	18,124
	(%)	2.3	2.4	1.1	2.5	3.0	1.7	3.4	1.9	2.8	1.9	3.7

Estimates of any use of psychiatric and of hospital mental health care were highest for immigrants from Middle East and North Africa (psychiatric care: males: 5.8 %, females: 7.6 %; hospital care: males: 2.1 %; females: 3.4) and lowest for persons from East Asian and Pacific (psychiatric care: males: 1.1 %, females: 1.5 %; hospital care: males: 0.5 % females: 1.1 %, Table 3). LTRs used more of both care types than most immigration region groups (psychiatry care: LTR males: 5.7 %, LTR females: 7.6 %; hospital care: LTR males: 2.8 %, LTR females: 3.7 %). Across outcomes, percentages of people with any use were lower for males than females for both immigrants and LTRs.

### Adjusted analyses

#### Mental health primary care

**Fig. 1:**
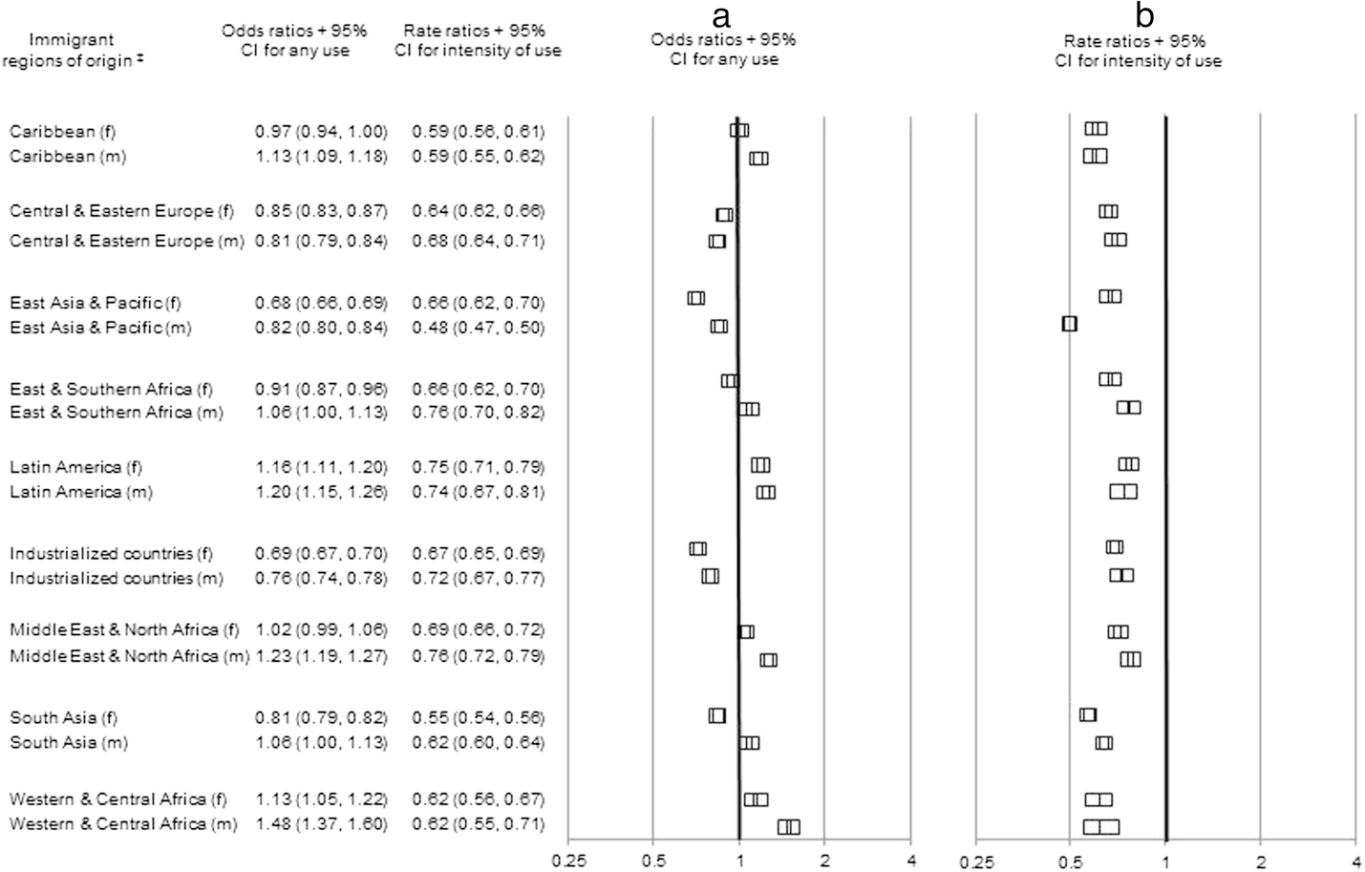
Odds ratios^†^ for any primary care visits (**a**) and rate ratios^†^ for the intensity of primary care visits (**b**) for non-psychotic disorders by adult immigrants within 5 years of arrival who settled in Ontario from 1993–2007 compared to their matched long term residents in urban Ontario. ^†^Odds ratios and rate ratios were determined from conditional logistic regression models and negative binomial regression models (respectively) stratified by immigrant region of origin and by sex. ^‡^Region of origin groupings were based on a modified version of the United Nations Children’s Fund (UNICEF) classification system

Estimates of any use of primary mental health care by immigrants compared to their matched LTRs varied by immigrant’s world region of origin. Likelihood of use was higher than LTRs for immigrants of both sexes from Western and Central Africa, Latin America, and for male newcomers from Caribbean, East and Southern Africa, Middle East and North Africa, and South Asia. Use was lower for both sexes from Central and Eastern Europe, East Asia and Pacific, and industrialized countries, and for females from East and South Africa, and from South Asia. The only differences from the unadjusted analysis were that females from Caribbean and Middle East and North Africa were higher than LTRs in the unadjusted analysis; this difference did not persist in the adjusted analysis. Among individuals with any primary care use, intensity of use was lower for immigrant region groups than for their LTR counterparts (Fig. 1).

### Psychiatric care

**Fig. 2:**
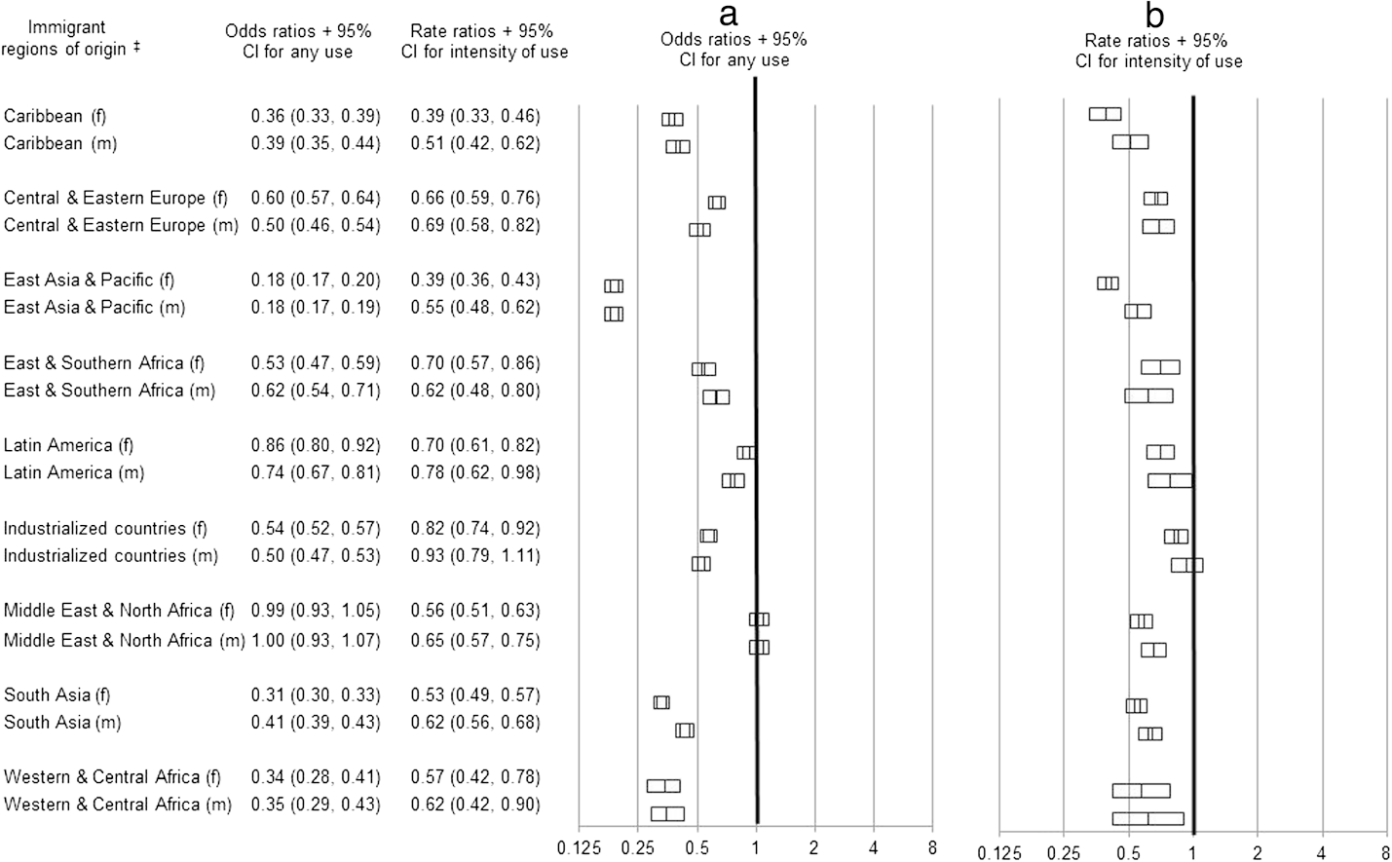
Odds ratios^†^ for any psychiatry visits (**a**) and rate ratios^†^ for the intensity of psychiatry visits (**b**) for non-psychotic disorders by adult immigrants within 5 years of arrival who settled in Ontario from 1993–2007 compared to their matched long term residents in urban Ontario. ^†^Odds ratios and rate ratios were determined from conditional logistic regression models and negative binomial regression models (respectively) stratified by immigrant region of origin and by sex. ^‡^Region of origin groupings were based on a modified version of the United Nations Children’s Fund (UNICEF) classification system

Newcomers were less likely to use any psychiatric care than their matched LTRs, except for immigrants from Middle East and North Africa whose estimates were not significantly different from their LTR comparators (Fig. 2). Immigrants also had lower intensity than LTRs, with one exception -- males from industrialized countries whose intensity of use estimate was not significantly different their comparators (Fig. 2).

### Mental health hospital care

**Fig. 3:**
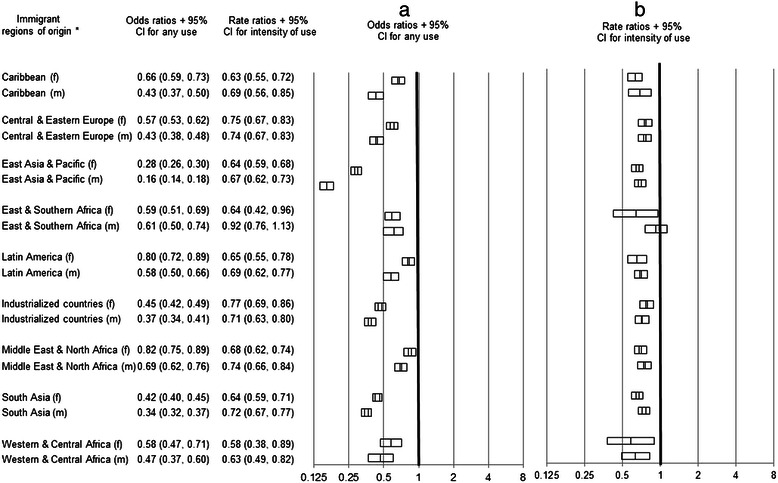
Odds ratios^†^ for any hospital use^‡^ (**a**) and rate ratios^†^ for the intensity of hospital use^‡^ (**b**) for non-psychotic disorders by adult immigrants within 5 years of arrival who settled in Ontario from 1993–2007 compared to their matched long term residents in urban Ontario. ^†^Odds ratios and rate ratios were determined from conditional logistic regression models and negative binomial regression models (respectively) stratified by immigrant region of origin and by sex. ^‡^Hospital use was defined as emergency department visits or admissions. *Region of origin groupings were based on a modified version of the United Nations Children’s Fund (UNICEF) classification system

Immigrants were significantly less likely than their matched LTRs to have any mental health hospital use and had lower use, (Fig. 3) with one exception -- males from East and South Africa whose intensity of use was not significantly differently from their matched LTRs.

### Trends across services

Across mental health services, estimates of use were consistent in their positioning relative to other world region groups. This was the pattern for newcomers from Middle East and North Africa who had higher estimates (ranging from 0.65 to 1.23 for males, and 0.56 to 1.02 for females), and for newcomers from East Asian and Pacific who had lower estimates (ranging from 0.16 to 0.82 for males, and 0.18 to 0.68 for females).

Sensitivity analyses showed that results were largely consistent with the primary analysis (See Additional file 4).

## Discussion

This population-based cross-sectional study examined mental health service by a heterogeneous population of recent newcomers representing all the major world regions. Descriptive data showed diverse immigrant profiles across the nine source regions for a number of characteristics that can affect service use, such as English language proficiency and visa admission class of entry. Patterns of mental health service use also differed by region, but showed that immigrants in Ontario from all world regions used less than their matched LTRs, especially for specialty mental health services.

Lower rates of mental health service use for newcomers have also been observed in other research [16, 45–48]. One possible explanation for this finding is the healthy immigrant effect, which states that newly arrived immigrants exhibit (general and mental) health advantages over native-born persons [49–54]. The healthy immigrant effect is likely due to multiple factors, including self-selection and screening prior to arrival. Selective migration has been raised as an explanation for the superior mental health of recent immigrants for almost one century [55, 56].

For many immigrant groups and LTRs we found similar rates of initial contact with primary mental health care. This may be because in many countries primary care is the main contact for mental health services, as is the case in Ontario [57, 58]. However, we also found that continued use of primary care was lower for almost all immigrant groups than LTRs. This may reflect that immigrants are more likely than others to become disengaged with western health services, perhaps due to culturally insensitive services, perceived over-willingness of physicians to provide pharmaceutical interventions, or recollections of physicians having a dismissive attitude and limited time in previous encounters [45, 59, 60]. Early discontinuation of primary care services warrants attention since it may reduce the likelihood of patients’ needs being addressed in service that is the recommended contact point for mental health care in Ontario and other jurisdictions [61, 62].

In contrast to primary care, in many countries specialty mental health services are minimally available [57, 63]. Lack of familiarity with specialty mental health services as they are delivered in Ontario may have contributed to the consistently lower use of specialty mental health care [64]. The exception was immigrants from industrialized countries (e.g., Australia, Denmark, England, France, New Zealand, etc.) who may be more accustomed to navigating mental health care systems that resemble those in Ontario (e.g., where insured mental health care is available following referral from primary care physicians who are the gate-keepers to specialized care) [64, 65]. This familiarity may explain why newcomers from industrialized countries generally had higher estimates of intensity of use of these services. In fact, males from this region were the only immigrant group whose use estimates were not different from LTRs. These findings support a need to reduce health disparities among immigrants by engaging in active efforts to clarify the role of mental health services to those unfamiliar with such services, [16, 60] especially since higher rates of initial contact with primary care services by some immigrant groups were not sustained.

In addition to variation in use related to type of service, patterns of use also varied depending on world region of origin. For example, newcomers from East Asia and Pacific showed relatively low estimates of use compared to LTRs, and those from Middle East and North Africa showed relatively high estimates of use. Within each region, a number of underlying system and personal factors may account for these results.

Regarding newcomers from Middle East and North Africa, other research has linked cultural practices and beliefs (e.g., health beliefs) to lower use in Asian immigrants [20, 46, 47, 66, 67]. Common beliefs in the Korean and Chinese communities that mental health disorders are Western problems and demonstrate weakness may inhibit expressions of illness and help seeking [57, 68, 69]. Corollaries of these beliefs are that newcomers from the East Asian and Pacific region may fear of being stigmatized by using Western health services and rely on informal support from strong familial and social networks [46, 70, 71]. Present data showed lower rates of speaking English and French among immigrants from East, Asia and Pacific. This may also have impeded help-seeking by this region group. Limited English proficiency can contribute to less satisfaction and a reduced likelihood of following recommendations for treatment and follow-up visits [72–75]. Another possible contributor to lower use by East Asian and Pacific newcomers is that in many countries in this source region the availability of specialty mental health care is limited, which may contribute to a lesser interest and familiarity with accessing speciality mental health services. In China, the ratio of psychiatrists per population is one-ninth of the ratio in Ontario [76]. There is almost certainly variation in mental health service use patterns among newcomers from different countries in the same source regions, although this could not be measured. Finally, individuals from East, Asia and Pacific were more commonly admitted in the economic class, which has stringent entrance criteria linked to health and potential to contribute to the host country economy; this may have contributed to lower mental health need [77].

In contrast to patterns observed among newcomers from East Asia and Pacific, this study found relatively high estimates of use of almost all services for immigrants from Middle East and North Africa. We speculate the reasons for this novel finding, since to the authors’ knowledge, no other potential reasons have been explored. Higher service use may reflect greater mental health need due to exposure to resettlement stressors (e.g., discrimination, unemployment) that appear more pronounced for this immigrant group. For example, Canadian unemployment rates for recent immigrants from Africa and the Middle East were higher than these rates for recent newcomers from other regions [78, 79]. High levels of English or French language proficiency for this group may also have enabled access to care. Finally, persons from Middle East and North Africa may have higher needs because as our data indicate, individuals from this region were more commonly admitted as refugees than in other admission classes. Admission as a refugee is associated with the most lenient entrance criteria, permitting entrants to have greater mental health need at arrival [77, 80]. Also, relative to other newcomers, refugees more commonly arrive as forced migrants who have had traumatic exposures, contributing to elevated rates of non-psychotic disorders, such as post-traumatic stress disorder [10, 81–83]. Admission in this class has been linked to more mental health service use [77]. Further investigation of prominent features and experiences among immigrants from varied source countries within the Middle East and North Africa region may help flag areas of potential vulnerability and contributors to high service use.

Present findings of heterogeneity in mental health service use among immigrants from different source regions aligns with research on general health disorders that has shown that both disparate health profiles [49, 50, 52, 84–90] and disparate health service use [91–93] among immigrants with different origins.

### Strengths and limitations

This study takes advantage of linked provincial health service and immigration databases in a setting with a high portion of diverse newcomers. This linkage allowed for the examination of use of different types of mental health services for newcomers from the main wold regions compared to matched long term residents in the same setting. Theoretical frameworks [10, 13, 94] and research on samples of immigrants [14, 15, 83] acknowledge the far-reaching consequences of the pre-migration context on health and social factors related to mental health service use. They have noted potential drivers of differences across region groups (e.g., economy in the source region, family structure, ethnicity, etc.). However, to the authors’ knowledge, no empirical studies have systematically examined immigrants from the full range of source countries represented in a population. Our work described the differences among broad world regions by comparing them to standardized non-recent immigrant comparators. World region of origin is likely a proxy measure for the plethora of pre-migration factors that influence use, [95] and its many underlying individual level factors need to be considered to make services more responsive to need.

The study also had a number of limitations. Given the numerous possible countries of origin, this study grouped people from geographically proximate regions together. This was done since these immigrants likely shared similar cultural and other characteristics that can affect service need and use. However, heterogeneity remained within groups and these region groupings did not allow for the examination of intragroup differences, or the identification of underlying factors that contribute to patterns of mental health service use observed for immigrants from each world region [96]. Elucidating the drivers of service use patterns for newcomers is important since Canadian immigration policy and other factors contribute to variation in regional immigration patterns over time.

Another limitation is that while the CIC contains information usually not available in health service databases, some desired information (e.g., mental health need, ethnicity, or use of alternative supports such as traditional folk medicine) [60] was not available. Since service use does not correspond to need, without further data we do not know if more limited use of services by immigrant region groups was linked to more unmet need, or no need for further services [16]. In addition, since this study focused on immigrants and comparators in the general population to help provide a meaningful comparison. Given this approach, analyses could not examine the impact of immigration related variables on immigrant mental health service use that likely accounted for the differences in service use related to world region of origin to elucidate drivers of observed patterns.

Similarly, data on use of Community Health Centres (CHCs) in Ontario could not be included. Although CHC clients have direct access to mental health community-based services without physician referrals, since CHCs serve a relatively small proportion of the Ontario immigrant population (1.4 %), [97] their exclusion likely did not significantly bias results.

The study also did not include immigrants who entered Ontario from a different province; refugee claimants who had not been accepted or were appealing; other temporary residents/workers/visitors; or ‘non-status’ residents. By erroneously attributing mental health care use by immigrants who were not included to LTRs, this study could have been biased against finding differences between immigrants and LTRs. However, the large sample of immigrants included in this study and the smaller relative size of excluded newcomers [98] suggests that results were not strongly affected by this limitation.

Finally, the study’s cross-sectional design was a limitation. Since we examined mental health care use during a snapshot in time rather than following immigrants across time, we could not establish causation between world region and mental health service use.

## Conclusion

This study used linked population-based administrative databases to examine the mental health service use by immigrants from the full spectrum of world regions living in a diverse Canadian province with universal health insurance. It found that immigrants from all world regions used fewer services for non-psychotic mental health disorders than LTRs, with the exception of primary mental health care, which immigrants were more or less likely to use than LTRs depending on their world region of origin. These results and similar findings help to combat stereotypes that newcomers over-use publicly funded services, including mental health services [99]. Future studies that examine mental health need and barriers to care, as well as other immigration specific factors, could begin to delineate the underlying reasons for patterns displayed by newcomers from various world regions of origin. Illuminating these underlying factors and how they relate to service use may help clinicians and planners determine if and how services should be targeted to meet the unique needs, norms, attitudes and knowledge of diverse immigrants in a multi-cultural context like Ontario, Canada.

## Additional files

Additional file 1: **Variables derived from the census.** (DOC 23 kb)
Additional file 2: **Region of origin classification groups.** (DOC 34 kb)
Additional file 3: **OHIP Diagnostic Codes (International Classification of Disease-9) Non Psychotic Mental Health Disorders.** (DOC 22 kb)
Additional file 4: **Results from the sensitivity analysis.** (DOC 41 kb)

## Abbreviations

CIC: Citizenship and Immigration Canada
ED: Emergency Department
LTR: Long term resident
OHIP: Ontario Health Insurance Plan
RPDB: Registered Persons Database
UNICEF: United Nations Children’s Fund
