# Supplementary material for: Mental health service use by recent immigrants from different world regions and by non-immigrants in Ontario, Canada: a cross-sectional study
Source: BMC Health Serv Res. 2015 Aug 20;15:336. doi: 10.1186/s12913-015-0995-9 (PMC4546085; doi:10.1186/s12913-015-0995-9)
Supplement: Additional file 1: — Variables derived from the census. (DOC 23 kb) [file 12913_2015_995_MOESM1_ESM.doc]

### Additional file 1: Variables derived from the census

Urban-rural designation (Statistics Canada)

Census data were provided by Statistics Canada. The census takes place every five years in Canada and is a reliable source of information for population and dwelling counts, as well as demographic and other socioeconomic characteristics. This study used Statistics Canada urban-rural variables.

Area-level urban versus rural designations. Statistics Canada’s methodology establishes an urban-rural dichotomy for Canada using methodology based on population size and density. An urban area was defined as having a population of at least 1,000 and a density of 400 or more people per square kilometre. All territory outside an urban area was defined as rural area. Together, urban and rural areas covered the entire nation. [25]

Area level income quintile (Statistics Canada)

Income quintile is a measure of relative household income adjusted for household size and community. Roughly 20% of Ontarians fall into each income quintile, with quintile 1 having the lowest income and quintile 5 the highest. Income quintile was derived using the Postal Code Conversion File Plus (PCCF+) to link the six-digit postal code of residence to census data at the smallest possible level (dissemination area or enumeration area). These estimates of household income were turned into estimates of neighbourhood income quintiles by pooling average income estimates for single-person equivalent, which yielded population quintiles by neighbourhood income for each dissemination area. [26]
