# Supplementary material for: Mental health service use by recent immigrants from different world regions and by non-immigrants in Ontario, Canada: a cross-sectional study
Source: BMC Health Serv Res. 2015 Aug 20;15:336. doi: 10.1186/s12913-015-0995-9 (PMC4546085; doi:10.1186/s12913-015-0995-9)
Supplement: Additional file 2: — Region of origin classification groups. (DOC 34 kb) [file 12913_2015_995_MOESM2_ESM.doc]

### Additional file 2: Region of origin classification groups

| **Industrialized countries**  Andorra  Australia  Austria  Belgium  Canada  Czech Republic  Denmark  Estonia  Finland  France  Germany  Greece  Hong Kong, China  Hungary  Iceland  Ireland  Israel  Italy  Japan  Lithuania  Luxembourg  Netherlands  New Zealand  Norway  Poland  Portugal  Republic of Korea  San Marino  Slovakia  Slovenia  Spain  Sweden  Switzerland  Turkey  United Kingdom  United States of America | **Central & East Europe**  Albania  Armenia  Azerbaijan  Belarus  Bosnia and Herzegovina  Bulgaria  Croatia  Georgia  Kazakhstan  Kyrgyzstan  Macedonia (The former Yugoslav Republic of)  Moldova  Montenegro  Romania  Russian Federation  Serbia  Tajikistan  Turkey  Turkmenistan  Ukraine  Uzbekistan | **Middle East & North Africa**  Algeria  Bahrain  Djibouti  Egypt  Iran (Islamic Republic of)  Iraq  Jordan  Kuwait  Lebanon  Libya  Morocco  Occupied Palestinian territory  Oman  Qatar  Saudi Arabia  Sudan  Syrian Arab Republic  Tunisia  United Arab Emirates  Yemen |
| --- | --- | --- |
| ***Eastern and Southern Africa***  Angola  Botswana  Burundi  Comoros  Eritrea  Ethiopia  Kenya  Lesotho  Madagascar  Malawi  Mozambique  Namibia  Rwanda  Seychelles  Somalia  South Africa  Swaziland  Tanzania, United Republic of  Uganda  Zambia  Zimbabwe | ***West and Central Africa***  Benin  Burkina Faso  Cameroon  Cape Verde  Central African Republic  Chad  Congo  Congo, Democratic Republic of the  Côte d'Ivoire  Equatorial Guinea  Gabon  Gambia  Ghana  Guinea  Guinea-Bissau  Liberia  Mali  Mauritania  Niger  Nigeria  Sao Tome and Principe  Senegal  Sierra Leone  Togo | **Caribbean**  Antigua and Barbuda  Barbados  Belize  British Virgin Islands  Dominica  Grenada  Guyana  Haiti  Jamaica  Montserrat  Saint Kitts and Nevis  Saint Lucia  Saint Vincent and the Grenadines  Suriname  Trinidad and Tobago  Turks and Caicos Islands |
| **Latin America**  Argentina  Bolivia  Brazil  Chile  Colombia  Costa Rica  Cuba  Dominican Republic  Ecuador  El Salvador  Guatemala  Honduras  Mexico  Nicaragua  Panama  Paraguay  Peru  Uruguay  Venezuela (Bolivarian Republic of) | **East Asia & Pacific**  Cambodia  China  Cook Islands  Fiji  Indonesia  Kiribati  Korea, Democratic People's Republic of  Lao People's Democratic Republic  Malaysia  Marshall Islands  Micronesia (Federated States of)  Mongolia Myanmar  Nauru  Niue  Palau  Papua New Guinea  Philippines  Samoa  Solomon Islands  Thailand  Timor-Leste  Tokelau  Tonga  Tuvalu  Vanuatu  Viet Nam | **South Asia**  Afghanistan  Bangladesh  Bhutan  India  Maldives  Nepal  Pakistan  Sri Lanka |
