# Supplementary material for: Mental health service use by recent immigrants from different world regions and by non-immigrants in Ontario, Canada: a cross-sectional study
Source: BMC Health Serv Res. 2015 Aug 20;15:336. doi: 10.1186/s12913-015-0995-9 (PMC4546085; doi:10.1186/s12913-015-0995-9)
Supplement: Additional file 3: — OHIP Diagnostic Codes (International Classification of Disease-9) Non Psychotic Mental Health Disorders. (DOC 22 kb) [file 12913_2015_995_MOESM3_ESM.doc]

**Additional file 3:**

**OHIP Diagnostic Codes (International Classification of Disease-9) Non Psychotic Mental Health Disorders**

anxiety neurosis, hysteria, neurasthenia, obsessive – compulsive neurosis, reactive depression, personality disorders, sexual deviations, alcoholism, drug dependence, psychosomatic illness, tics, anorexia nervosa adjustment reaction, depressive disorder, economic problems, marital difficulties, parent- child problems, problems with aged parents or in-laws, family disruption/ divorce, education problems, ilegitimacy, social maladjustment, occupational problems, legal problems other problems of social adjustment
