# Supplementary material for: Mental health service use by recent immigrants from different world regions and by non-immigrants in Ontario, Canada: a cross-sectional study
Source: BMC Health Serv Res. 2015 Aug 20;15:336. doi: 10.1186/s12913-015-0995-9 (PMC4546085; doi:10.1186/s12913-015-0995-9)
Supplement: Additional file 4: — Results from the sensitivity analysis. (DOC 41 kb) [file 12913_2015_995_MOESM4_ESM.doc]

**Additional file 4: Results from the sensitivity analysis**

| **Table 1.** Estimates and 95% confidence intervals from the sensitivity analysis looking at hospital use for the most responsible diagnosis by world region and sex groups. Results that differed from the primary analysis are presented in this table. | | | | |
| --- | --- | --- | --- | --- |
| **Odds ratios for any use** | | | | |
|  | **Sensitivity analysis** | **Primary analysis** | **Sensitivity analysis** | **Primary analysis** |
| **Worldwide region** | **Males** | | **Females** | |
| Caribbean | Not different ^†^ | Not different ^†^ | Not different ^†^ | Not different ^†^ |
| Central and Eastern Europe | Not different ^†^ | Not different ^†^ | Not different ^†^ | Not different ^†^ |
| East Asia and Pacific | Not different ^†^ | Not different ^†^ | Not different ^†^ | Not different ^†^ |
| East and Southern Africa | Not different ^†^ | Not different ^†^ | Not different ^†^ | Not different ^†^ |
| Latin America | Not different ^†^ | Not different ^†^ | 0.87 (0.74,1.03) | 0.80 (0.72,0.89) |
| Industrialized countries | Not different ^†^ | Not different ^†^ | Not different ^†^ | Not different ^†^ |
| Middle East and North Africa | Not different ^†^ | Not different ^†^ | Not different ^†^ | Not different ^†^ |
| South Asia | Not different ^†^ | Not different ^†^ | Not different ^†^ | Not different ^†^ |
| West and Central Africa | Not different ^†^ | Not different ^†^ | 0.73 (0.53,1.00) | 0.58 (0.47,0.71) |
| **Rate ratios for counts of use** | | | | |
|  | **Males** | | **Females** | |
| Caribbean | Not different ^†^ | Not different ^†^ | Not different ^†^ | Not different ^†^ |
| Central and Eastern Europe | Not different ^†^ | Not different ^†^ | Not different ^†^ | Not different ^†^ |
| East Asia and Pacific | Not different ^†^ | Not different ^†^ | Not different ^†^ | Not different ^†^ |
| East and Southern Africa | Not different ^†^ | Not different ^†^ | 0.57 (0.29, 1.14) | 0.64 (0.42, 0.96) |
| Latin America | Not different ^†^ | Not different ^†^ | 0.75 (0.63, 0.88) | 0.65 (0.55, 0.78) |
| Industrialized countries | Not different ^†^ | Not different ^†^ | Not different ^†^ | Not different ^†^ |
| Middle East and North Africa | 0.70 (0.60,0.84) | 0.74 (0.66, 0.84) | Not different ^†^ | Not different ^†^ |
| South Asia | Not different ^†^ | Not different ^†^ | Not different ^†^ | Not different ^†^ |
| West and Central Africa | Not different ^†^ | Not different ^†^ | Not different ^†^ | Not different ^†^ |
| ^†^ The estimates and confidence limits from the sensitivity analysis were in the same direction (above or below 1 or not significant) as the primary analysis | | | | |
